# CDCA2 Inhibits Apoptosis and Promotes Cell Proliferation in Prostate Cancer and Is Directly Regulated by HIF-1α Pathway

**DOI:** 10.3389/fonc.2020.00725

**Published:** 2020-05-19

**Authors:** Yixiang Zhang, Yingduan Cheng, Zhaoxia Zhang, Zhongyuan Bai, Hongtao Jin, Xiaojing Guo, Xiaoyan Huang, Meiqi Li, Maolin Wang, Xing-sheng Shu, Yeqing Yuan, Ying Ying

**Affiliations:** ^1^Department of Urology, Shenzhen People's Hospital, The Second Clinical Medical College of Jinan University, The First Affiliated Hospital of Southern University of Science and Technology, Shenzhen, China; ^2^Department of Translational Molecular Medicine, Saint John's Health Center, John Wayne Cancer Institute, PHS, Santa Monica, CA, United States; ^3^Department of Pediatrics, Shenzhen People's Hospital, The Second Clinical Medical College of Jinan University, The First Affiliated Hospital of Southern University of Science and Technology, Shenzhen, China; ^4^Department of Pathology, Shenzhen People's Hospital, The Second Clinical Medical College of Jinan University, The First Affiliated Hospital of Southern University of Science and Technology, Shenzhen, China; ^5^Department of Physiology, School of Basic Medical Sciences, Shenzhen University Health Sciences Center, Shenzhen University, Shenzhen, China

**Keywords:** CDCA2, prostate cancer, apoptosis, proliferation, HIF-1a

## Abstract

Prostate cancer (PCa) is a major serious malignant tumor and is commonly diagnosed in older men. Identification of novel cancer-related genes in PCa is important for understanding its tumorigenesis mechanism and developing new therapies against PCa. Here, we used RNA sequencing to identify the specific genes, which are upregulated in PCa cell lines and tissues. The cell division cycle associated protein (CDCA) family, which plays a critical role in cell division and proliferation, is upregulated in the PCa cell lines of our RNA-Sequencing data. Moreover, we found that *CDCA2* is overexpressed, and its protein level positively correlates with its histological grade, clinical stage, and Gleason Score. CDCA2 was further found to be upregulated and correlated with poor prognosis and patient survival in multiple cancer types in The Cancer Genome Atlas (TCGA) dataset. The functional study suggests that inhibition of CDCA2 will lead to apoptosis and lower proliferation *in vitro*. Silencing of CDCA2 also repressed tumor growth *in vivo*. Loss of CDCA2 affects several oncogenic pathways, including MAPK signaling. In addition, we further demonstrated that *CDCA2* was induced in hypoxia and directly regulated by the HIF-1α/Smad3 complex. Thus, our data indicate that CDCA2 could act as an oncogene and is regulated by hypoxia and the HIF-1αpathway. *CDCA2* may be a useful prognostic biomarker and potential therapeutic target for PCa.

## Introduction

In the United States, prostate cancer (PCa) is the second leading cause of cancer death in men. In 2017 in the United States, about 161,360 people were diagnosed as PCa, and, of these, 26,730 PCa patients died (1). Although the treatment has made significant progress, around one third of organ-confined PCa patients who underwent surgical treatment would progressed to aggressive or metastatic PCa within 10 years (2). Thus, it is important to elucidate the PCa tumorigenic mechanism by investigating PCa-related genes, which will help provide better therapeutic strategies for treatment.

Increasing lines of evidence have suggested that cell division cycle associated proteins (CDCA) are critical in tumor progression. CDCA1 is a promising prognostic tissue biomarker in oral cavity cancer (3). Overexpression of CDCA2 and CDCA3 is a frequent event for oral squamous cell carcinomas (OSCC). Their overexpression would prevent G1 arrest through repressing cyclin-dependent kinase inhibitors and inducing apoptosis (4, 5). CDCA4 enhances proliferation and reduces the cellular apoptotic rate in human breast cancer MCF-7/ADM cells (6). CDCA5is overexpressed and functions as an oncogene in bladder cancer, hepatocellular carcinoma, and gastric cancer (7–9). In addition, CDCA3 and CDCA5 mRNA expression levels are significantly elevated in breast tumor samples or breast cancer cells compared with normal controls (10). So far, the expression and role of the CDCA family in the prostate is not sufficiently studied.

Pathological hypoxia is critical in the process of cancer progression and dissemination, and it also affects both tumor microenvironment and cancer cells. In addition, hypoxia also plays a vital role in epithelial to mesenchymal transition (EMT)-like cancer cell migration and drug resistance (11). In hypoxic condition, several pathways, including HIF, PI3K, MAPK, and NFκB pathways, will interact with each other, lead positive and negative feedback loops, and will strengthen or attenuate hypoxic effects (11). Hypoxic conditions in PCa are associated with poor prognosis (12, 13). Under hypoxia stimulation, genes induced by HIF, PI3K, MAPK, or NFκB pathways may contribute to the cancer prognosis. For example, choline kinase (Chk) contributes to malignant transformation and progression of prostate cancer and is controlled by HIF-1α signaling (14). In prostate cancer, the HIF-1α is expression regulator of S100A8/A9, which play important roles during tumorigenesis, affecting inflammatory processes, proliferation, invasion, and metastasis of tumor cells (15). HIF-1α also directly regulates BCL-xL, which is a hypoxia-responsive, anti-apoptotic protein of the Bcl-2 family and is also overexpressed in prostate carcinoma and many other cancers (16). In some conditions, HIF-1α can directly interact with Smad3 and synergistically regulate hypoxia targets expression (17, 18).

Of interest, we found that majority of the genes encoding for the *CDCA* family were upregulated in PCa, as confirmed by RNA sequencing data. Among this family, CDCA2, also named as Repo-Man (Recruits PP1 onto Mitotic chromatinat anaphase), is a nuclear protein that binds to protein phosphatase 1 γ (PP1γ). CDCA2 is responsible for the targeting of PP1 to chromatin during anaphase, leading to the dephosphorylation of H3 and controls cell proliferation *in vitro* (19, 20). But its function in cancer, especially in prostate cancer, is not well defined. In this study, we chose CDCA2 to further investigate its function in PCa. Our result suggests that CDCA2 was overexpressed in PCa and many other cancer types. It functions as an oncogene in PCa, as confirmed in both *in vitro* and *in vivo* studies. We also found CDCA2 is induced in hypoxia condition and directly regulated by HIF-1α. This gene may represent a novel and useful prognostic biomarker and therapeutic biomarker for PCa.

## Materials and Methods

### Cell Culture and Transfection

A series of PCa cell lines (LAPC4, 22RV1, LNCap, Du145, and PC3) were used in this study. In brief, PCa cell lines were maintained in RPMI 1640 medium (Invitrogen, Carlsbad, CA) supplemented with 10% fetal bovine serum (FBS), 1% penicillin-streptomycin (10 ng/ml penicillin and 10 U/ml streptomycin), and 2.5 mM glutamine at 37°C in a humidified 5% CO_2_ incubator. Control siRNA (siControl) and CDCA2 short interfering RNA (siCDCA2), which were ordered from Santa Cruz Biotechnology (Dallas, TX, USA), were transfected with an RNAiMAX transfection reagent (Invitrogen, Eugene, OR, USA).

### RNA Sequencing

RNAeasy Mini kit (QIAGEN) was used for high quality RNA extraction. Normal prostate RNA was purchased from Clontech (Clontech, Palo Alto, CA). An RNA Sequencing library was prepared according to the KAPA biosystems' manual. The prepared libraries were analyzed by Illumina HiSeq platform. The RNA sequencing reads were analyzed by using the standard protocol as previously reported (21).

### Reverse Transcription and Quantitative Real-Time PCR

Reverse transcription was performed according to manual of manufacturers (Invitrogen, Eugene, OR, USA). In brief, 1 μg of RNA was used for each reverse transcription reaction. Quantitative real-time PCR was carried out with SYBR Green master mix in Applied Biosystems 7,300 Real-time systems. All Real-time PCR primers used are listed in Supplemental Table 1 Each experiment was performed in three independent experiments.

### Cell Proliferation Assay

Du145 and PC3 cells were used for growth curve study. In brief, cells were seeded in six-well plates at an appropriate density without penicillin/streptomycin. After 16-h, cells were then transfected with siControlor CDCA2 siRNA, respectively. Then cells were trypsinized and collected for cell number counting at 0-, 24-, and 48-h time points after transfection. Each experiment was repeated at least three times.

### Apoptosis Assay

PE Annexin V Apoptosis Detection Kit I (BD Bioscience) was used for apoptotic cells detection by flow cytometry. About 48-h after transfection, cells were harvested and rinsed with cold PBS once. Then cells were suspended in a binding buffer, and about 1 × 10^5^cells were used for staining with PE Annexin V and 7-AAD. After 15-min incubation at room temperature in the dark, stained cells were then analyzed by flow cytometry. The apoptotic percentage was calculated as the sum of early apoptotic cells and late apoptotic cells. Each experiment was conducted in triplicate in three independent experiments.

### Construction of CDCA2 Knock-Down Stable Cell Lines

The lenti-virus coding for short-hairpins RNA (shRNA) targeting the CDCA2 region (5'-GUCUGUGGCAAGAGGGAAA-3') was obtained from GENECHEM (Shanghai, China). A total of 1 × 10^5^ Du145 cells were infected with a total volume of 100 μl of lenti-virus for 24-h. Cells that expressed the targeted lentiviral shRNAs (shCDCA2-Du145) or scramble control (shControl-Du145) were obtained by selecting the infected cells with 1 mg/mL of puromycin for 3 to 4 weeks at 37°C.

### Tumor Xenograft Assay

Animal experiments and maintenance were approved by the Laboratory Animal Ethics Committee of Shenzhen University. Twelve BALB/c nude mice (6 weeks old) were injected subcutaneously with 2 × 10^6^ shControl-Du145 or shCDCA2-Du145 cells per animal. The body weight and tumor diameters of the mice were measured every 3 or 4 days. Tumor size was measured every other day with calipers, and tumor volumes were calculated using a formula: volume = (width)^2^× length/2. All of the infected mice were dissected 6 weeks later, and their tumors were weighed.

### Oncomine Analysis

Two Oncomine prostate-cancer-related microarray databases were used in the expression profile, and the CDCA2 expression profile in PCa was further validated in an Oncomine cancer microarray database (http://www.oncomine.com) (22). Two representative data groups were established (with more than 2-fold CDCA2 changes).

### The Cancer Genome Atlas (TCGA) Data Analysis

The expression levels of the CDCA family in PCa were analyzed using TCGA data. We searched Ualcan, which is an online portal for facilitating tumor subgroup gene expression based on TGCA data (Ualcan: http://ualcan.path.uab.edu/) (23). Their expression in normal and primary PCa tumors, and their expression based on the Gleason score (GS) was analyzed. In addition, we also analyzed the expression and prognosis information of CDCA2 in all other cancer types listed at Ualcan. CDCA2 genomic copy numbers in cancers was cited from TCGA Copy Number Portal (http://portals.broadinstitute.org/tcga/home) (24).

### Gene Expression Omnibus (GEO) Dataset Analysis

A set of ChIP-Sequencing and RNA-Sequencing datasets were downloaded from GEO. These included a hypoxia-related ChIP-Sequencing and RNA-Sequencing dataset in PCa PC3 cells (GSE106305) (25), a microarray for hypoxia- and normoxia-treated PCa PC3 cells (GSE53012 and GSE80657) (26, 27), a microarray for hypoxia- and normoxia-treated lung cancer A549 cells (GSE48134)(28), a microarray for HIF-1α inhibitor (echinomycin)-treated glioma U251 cells (GSE7835) (29), a microarray for HIF-1α knockdown breast cancer MCF7 cells (GSE3188), and a MEN1 ChIP-Sequencing dataset in prostate cancer (GSE132827**)** (30, 31). The method for ChIP-Sequencing data analysis was described before (32).

### Patient Samples and IHC

All benign prostatic hyperplasia (BPH) and PCa specimens were collected with the patients' consent in Shenzhen People's Hospital. The patients' tissues were fixed in 10 % buffered formalin and embedded in paraffin. CDCA2 antibody (abcam) was used for IHC staining of tissue samples. The IHC staining was performed as described previously (33). According to the 2010 AJCC Cancer Staging Manual seventh edition, tumor staging and differentiation of PCa was defined as 1) Well differentiation:Gleason Score ≤ 6; 2) Moderate differentiation: Gleason Score = 7; and 3) Poor differentiation:Gleason Score ≥ 8. Association between CDCA2 expression and clinicopathological characteristics were analyzed by Chi-squared and Fisher's exact test.

### Statistical Analysis

All data in this paper are presented as mean ± standard deviation. Statistical assessments were carried out using Student's *t*-test or one-way anova. *P* < 0.05 was considered statistically significant.

## Results

### The Cell Division Cycle Associated Protein Family Is Increased in Prostate Cancer

**Figure 1:**
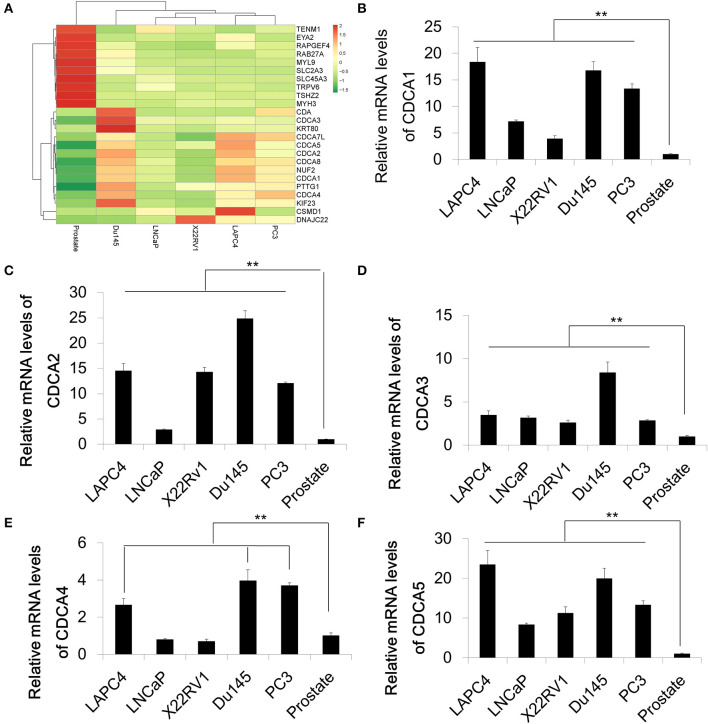
The cell division cycle associated protein (CDCA) family is increased in prostate cancer. **(A)** Heatmap of RNA sequencing results of a series of prostate cancer cells and normal prostate tissue. **(B–F)** Real-time PCR for validation of RAN-sequencing results for CDCA1 **(B)**, CDCA2 **(C)**, CDCA3 **(D)**, CDCA4, and CDCA5 **(F)**. All the data are shown as mean ± SD. **P < 0.01.

In order to identify differential expression in PCa, we used RNA-Sequencing to test the mRNA levels of Pca cell lines and normal prostate tissue. A series of Pca cell lines (LAPC4, 22RV1, LNCap, Du145, and PC3) were used in this study. The RNA of normal prostate tissue was used as a control. We compared the average expression levels in prostate cancer cells and normal prostate tissues (GEO accession number:GSE148544). The RNA sequencing data showed that genes coding for the cell division cycle associated protein (CDCA) family that contains *CDCA1* to *CDCA5* are upregulated in prostate cancer cell lines with different increasing levels (Figure 1A). The overexpression and oncogenic roles of CDCA family have been reported in several types of tumors but not in PCa. We then performed a quantitative Real-time PCR to verify the mRNA expression of some representative CDCA members: *CDCA1* to *CDCA5* (Figures 1B–F). Consistent with our RNA-sequencing results, the mRNA levels of *CDCA1* to *CDCA5* increased in most of PCa cell lines.

Among our identified CDCA family members, *CDCA2* is a well-studied gene involved in cell division. It is necessary for the establishment of heterochromatin in post-mitotic cells, and it is also required for nuclear envelope reassembly during mitotic exit (19, 20, 34). Previous studies also suggested that *CDCA2* was overexpressed in neuroblastoma, lung cancer, and oral squamous cell carcinoma (5, 35, 36), but few reports are related to *CDCA2* function in PCa. Here, we chose to study *CDCA2* further in PCa.

### CDCA2 Is Overexpressed in Primary PCa and Positively Correlated With Poor Prognosis in Patients With PCa

**Figure 2:**
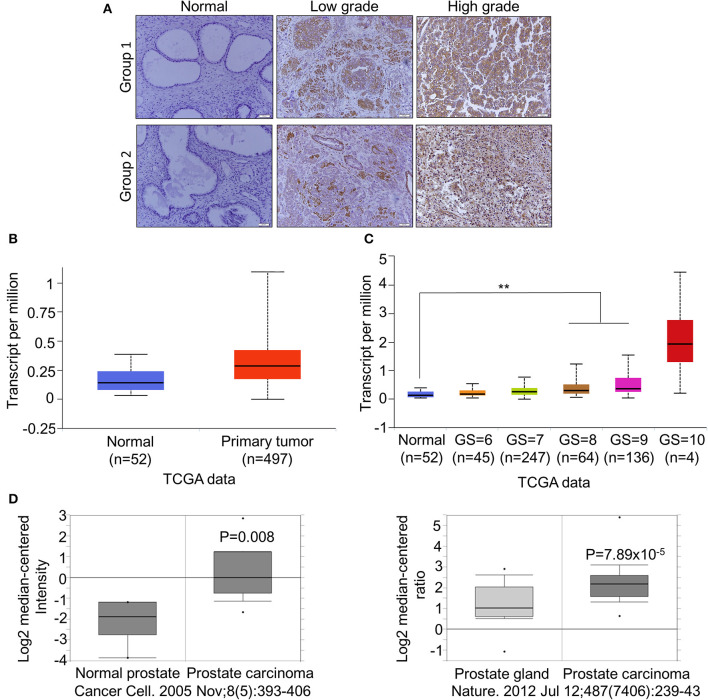
CDCA2 is overexpressed in primary PCa and positively correlated with poor prognosis in patients with PCa. **(A)** Representative images of CDCA2 IHC staining in normal prostate tissues and prostate cancer tissues. **(B)** Expression level of CDCA2 in TCGA prostate cancer study. Figure was cited from Ualcan. *P* = 0.0001. **(C)** Expression level of CDCA2 for different GS score cohorts in TCGA prostate cancer study. Figures were cited from Ualcan. **P < 0.01. **(D)** Expression levels of CDCA2 in some prostate cancer studies in Oncomine database. *P-*value was as indicated.

To further confirm the overexpression of CDCA2 in PCa, we checked the protein levels in primary PCa patients' samples. Ninety BPH and 90 PCa tumor samples were collected in Shenzhen Peoples' Hospital. CDCA2 antibody was used for immunohistochemistry (IHC) staining. The IHC staining results showed that CDCA2 was overexpressed in the prostate cancer tissues (Figure 2A). Our data indicated that CDCA2 was highly expressed in 58% (52/90) of PCa tumor samples but with only 24% (22/90) frequency in BPH (*p* = 0.0001) (Table 1). We further found that 63% (19/39) of patients with a Gleason Score (GS) ≤ 6 showed lower CDCA2 expression and 71% (20/28) of men with a GS of 8–10 displayed higher CDCA2 expression (*P* = 0.0392, Table 2). Our results indicated that the overexpression of CDCA2 was negatively correlated with PCa progression.

**Table 1:** Expression of cdca2 in BPH and PCa tissues.

**Type**	**No. of patients**	**Scores for cdca2 signal**				***P*-value**
		**Low expression**		**High expression**		
		**–**	**+**	**++**	**+++**	
PCa	90	5	33	33	19	0.0001
BPH	90	15	53	15	7	

**Table 2:** Relationship between clinicopathological variables and CDCA2 expression level in PCa patients.

**Classification**	**No. of patients**	**CDCA2 signal**				***P*-value**
		**–**	**+**	**++**	**+++**	
Age (years)						0.9020
>70	49	3	19	18	9	
≤ 70	41	2	14	15	10	
Gleason score						0.0392
≤ 6	39	3	16	15	5	
7	23	2	9	7	5	
≥8	28	0	8	11	9	
Recurrence						0.5110
Yes	27	2	8	0	7	
No	63	3	25	23	12	

We searched other groups' finding to further support our results. When compared with normal tissues, the mRNA levels of CDCA2 was significantly increased in patients with PCa, as found in a TCGA dataset provided by Ualcan (Figure 2B), and its expression was significantly positively correlated with GS except GS = 10, which is not surprising since the patient number of GS = 10 is too small (*n* = 4) (Figure 2C). We further examined the expression of CDCA2 in other cancer types. In all 33 TCGA cancer types, 18 of them exhibited significantly higher CDCA2 expression (Supplemental Table 2). And the number of cancer types is expected to be more than 18 since numbers of normal control are too small for some cancer types to get significant biostatical analysis. What is more, higher CDCA2 expression was correlated to patients' survival in some cancer types, such as in lung adenocarcinoma (LAUD), Kidney renal papillary carcinoma (KIRP), and (liver hepatocellular carcinoma) LIHC (Supplemental Figure 1). Besides TCGA data, we also investigated the Oncomine database for two particular studies and found that CDCA2 was increased in primary PCa by about 2.899-fold in Varambally's study and 1.889-fold in Grasso's study, when compared to a normal prostate gland (37, 38)(Figure 2D). All data clearly indicated that CDCA2 was overexpressed in PCa and is a candidate oncogene for PCa and multiple other cancers.

### Inhibition of CDCA2 Arrests Proliferation and Induces Apoptosis in PCa Cell Lines

**Figure 3:**
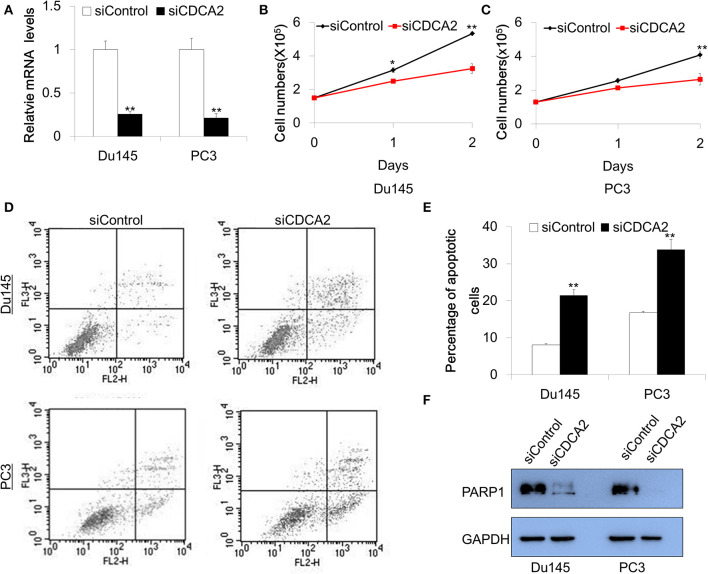
Knockdown of *CDCA2* inhibits proliferation and induces apoptosis in PCa cell lines. **(A)** Knockdown efficacy of CDCA2 siRNA in Du145 and PC3 prostate cancer cell lines. **P < 0.01. **(B)** Growth curve of Du145 for CDCA2 knockdown cells. *P < 0.05 and**P < 0.01. **(C)** Growth curve of PC3 for CDCA2 knockdown cells. **P < 0.01. **(D)** Representative figures of flow cytometry for apoptosis study. **(E)** Statistical results for apoptosis study in Du145 and PC3 cell lines. **(F)** Western blot for total PARP-1 level changes in Du145 and PC3 cells with CDCA2 inhibition by siRNA. All the data are shown as mean ± SD and **P < 0.01. All experiments were repeated for three times.

Our data—shown above—indicates that *CDCA2* is a potential oncogene in PCa. To explore the function of CDCA2 in PCa, we adopted a small interfering RNA (siRNA) to knockdown *CDCA2* and examined the effect of CDCA2 on cellular proliferation. Du145 and PC3 were chosen for functional study since they have higher *CDCA2* expression. Firstly, we checked the knockdown efficacy after 48-h transfection with siRNA. Quantitative Real-time PCR analysis showed that the knockdown efficacy was 75 and 79% in Du145 and PC3, respectively (Figure 3A). We counted the cell numbers after transfection and found that cellular proliferation rate was lower in *CDCA2* silenced cells (Figures 3B,C).

We further examined the effect of CDCA2 on apoptosis. Cells were stained with Annexin V-PE/7-AAD and analyzed by flow cytometry after 48-h siRNA transfection. We found that the inhibition of *CDCA2* increased the apoptotic cell numbers in Du145 and PC3 cells (Figures 3D,E). We also found that loss of CDCA2 reduced protein level of PARP-1 (Figure 3F). Collectively, our data indicate that CDCA2 plays a role in regulating cellular proliferation and apoptosis, which contributes to the tumorigenesis of PCa.

### CDCA2 Regulates Genes Involved in Cellular Proliferation and Apoptosis

**Figure 4:**
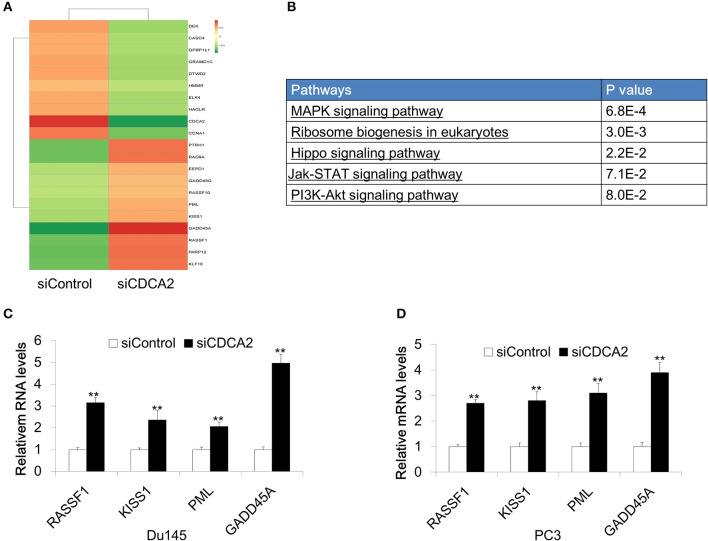
Identification of differentially expressed genes in Du145 cells upon CDCA2 repression. **(A)** Heatmap of RNA- sequencing results for CDCA2 knockdown Du145 cells. **(B)** Pathways affected by CDCA2 suppression were identified by KEGG pathways analysis. **(C,D)** Real-time PCR for some selected genes in Du145 and PC3 cells. All the data are shown as mean ± SD and **P < 0.01. All experiments were repeated for three times.

In order to further investigate the mechanism of CDCA2 in cellular proliferation and apoptosis, we performed RNA sequencing on *CDCA2-*silenced Du145 cells. About 2821 genes were changed with a 2-fold difference found in *CDCA2* silenced cells (Figure 4A) (GEO accession number:GSE148544). KEGG pathway analysis by online microarray analysis toolkit (https://david.ncifcrf.gov/) indicated that loss of CDCA2 affects the MAPK signaling pathway, ribosome biogenesis, Hippo pathway, Jak-STAT pathway, and PI3K-Akt pathway (Figure 4B). Except for this its role in signaling pathway, among the genes with 2-fold changes, we found some well-reported tumor suppressor genes were upregulated in *CDCA2* knockdown cells, including *RASSF1, KISS1, PML*, and *GADD45A*, as shown in Figure 4A. We further confirmed the gene expression levels of these genes by quantitative Real-time PCR both in Du145 and PC3 cells. The expression of the potential targeted genes was significantly increased after the inhibition of *CDCA2*, as shown in Figures 4C,D. Thus, the alteration of oncogenic pathway and upregulated tumor suppressors might play a role in induction of apoptosis and inhibition of cellular proliferation when *CDCA2* is repressed.

### Inhibition of CDCA2 Suppresses Tumor Growth *in vivo*

**Figure 5:**
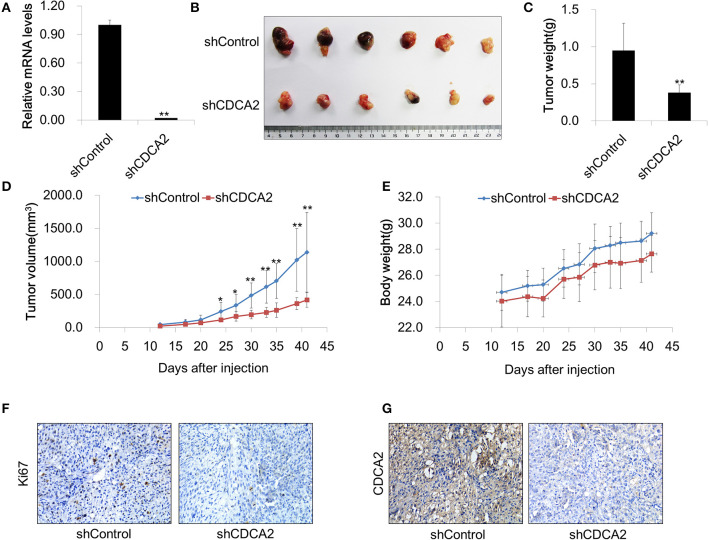
Inhibition of CDCA2 suppresses tumor growth *in vivo*. **(A)** Knockdown efficiency of CDCA2 shRNA stable cell line. **(B)** Representative images of the tumors isolated from the mice. **(C)** Weight of tumors. **(D)** Tumor volume measured at different time points. **(E)** Body weight measured at different time points. **(F,G)** Representative immunohistochemical staining images of Ki67 **(F)** and CDCA2 **(G)** in dissected tumor tissues. All the data are shown as mean ± SD.*P < 0.05 and **P < 0.01.

To further evaluate the function of CDCA2 *in vivo*, we determined whether knockdown of *CDCA2* could inhibit tumor xenograft growth in nude mice. To this end, BALB/c nude mice (6 weeks old) were injected subcutaneously with 2 × 10^6^ of Du145 shRNA scramble control (shControl-Du145) or *CDCA2* knockdown cells (shCDCA2-Du145) per mice and were dissected 6 weeks later. We found that knockdown of *CDCA2* inhibited tumor growth, leading to significantly reduced tumor volumes (Figures 5A–C) and mass (Figure 5D) but not body weight (Figure 5E). In addition, IHC staining for the proliferation marker Ki67 revealed that expression of Ki67 was negatively associated with CDCA2 in dissected tumor mass (Figures 5F,G), indicating that CDCA2 repression led to inhibition of tumor cell proliferation. Thus, our data demonstrated that knockdown of *CDCA2* inhibited tumor growth *in vivo*.

### Overexpression of CDCA2 Was Not Caused by Genomic Amplification

**Figure 6:**
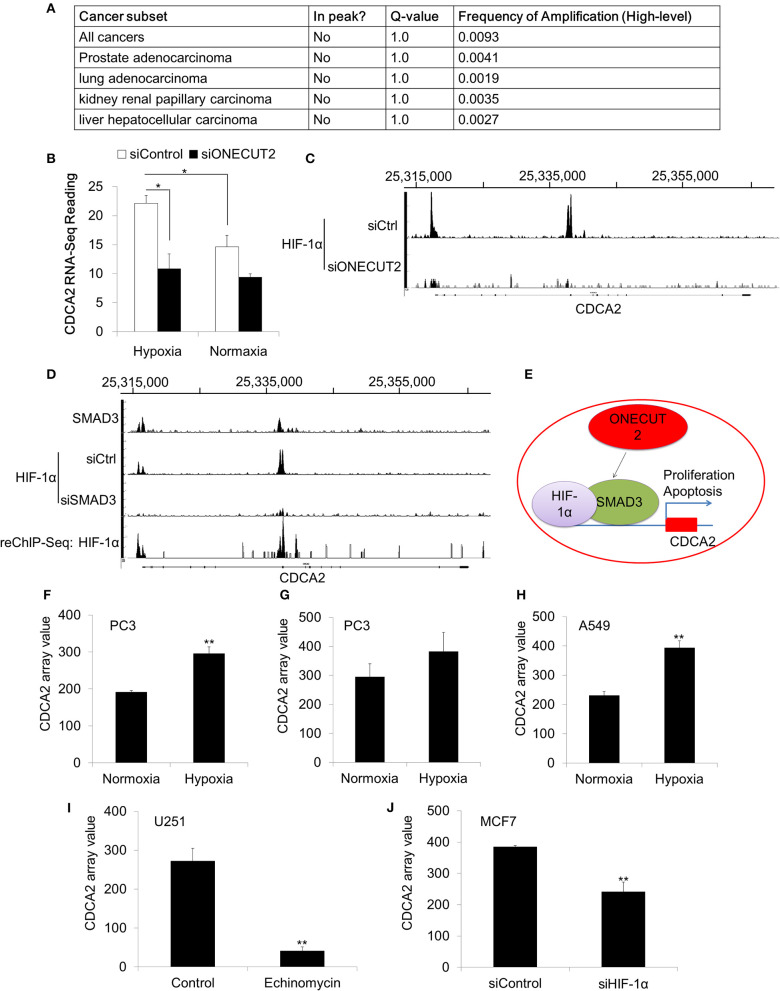
CDCA2 was regulated by the hypoxia signaling pathway in prostate cancer. **(A)** Genomic amplification analysis of CDCA2 in TCGA datasets. **(B)** RNA-Sequencing reading of CDCA2 in hypoxia and normoxia condition. **(C)** ChIP-Sequencing of HIF-1αon the promoter of CDCA2 at ONECUT2 deficiency. **(D)** SMAD3 ChIP-Sequencing, HIF-1α ChIP-Sequencing, and SMAD3-HIF-1α ChIP-re-ChIP-Sequencing on promoter regions of CDCA2. **(E)** Mechanism model of CDCA2 function and regulation in PCa. **(F)** CDCA2 array value in normoxia- and hypoxia-treated PC3 cells (GSE53012); **(G)** CDCA2 array value in normoxia- and hypoxia-treated PC3 cells (GSE80657); **(H)** CDCA2 array value in normoxia- and hypoxia-treated lung cancer A549 cells (GSE48134); **(I)** CDCA2 array value in HIF-1α inhibitor-enchinomycin-treated glioma U251 cells (GSE7835); **(J)** CDCA2 array value in HIF-1α knockdown breast cancer MCF7 cells (GSE3188). All the data are shown as mean ± SD. *P < 0.05, **P < 0.01.

Our data has proved that CDCA2 was overexpressed in PCa. We were interested in the mechanism of CDCA2 upregulation. We analyzed the TCGA dataset, which has the genomic sequencing data for multiple cancer types. To our surprise, no genomic amplification was found for CDCA2 in all 40 cancer types (TCGA Copy Number Portal, http://portals.broadinstitute.org/tcga/home) (Figure 6A) (24). This indicated that CDCA2 overexpression was controlled by transcriptional regulation.

### CDCA2 Was Induced in Hypoxic Condition and Regulated by the HIF-1αPathway

To further elucidate the overexpression mechanism of CDCA2 in PCa, we analyzed some published ChIP-Sequencing and RNA-Sequencing results. In a published ChIP-Sequencing and RNA-Sequencing dataset (GSE106305), we found CDCA2 was elevated in the hypoxia condition (Figure 6B). Hypoxia is strongly associated with PCa progression (39, 40). Due to the rapid growth of cancer cells in solid tumors, hypoxia often occurs in tumors and can cause tumors to change and adapt to the hypoxic condition. Overexpression of CDCA2 may be necessary for proliferation and adaptation to hypoxia in solid tumors.

Beside this, we also found CDCA2 was repressed by ONEUCT2 deficiency. It has been reported that ONEUCT2 drives tumor aggressiveness in neuroendocrine prostate cancer (40), partially through regulating tumor hypoxia and hypoxia signaling. Specifically, ONECUT2 activates SMAD3, which regulates hypoxia signaling through moderating HIF-1α transcriptional binding, causing higher degrees of hypoxia in NEPC compared to prostate adenocarcinomas (40). We found that CDCA2 was repressed at ONECUT2 deficiency, and this could be explained by lower HIF-1α binding to a CDCA2 promoter (Figures 6B,C). We further found that SMAD3 binds to a CDCA2 promoter to recruit HIF-1α, and its deficiency will lower HIF-1α binding on a CDCA2 promoter region; this was evidenced by SMAD3 and HIF-1α ChIP-re-ChIP (Figure 6D). Our data indicated that CDCA2 is a target of the hypoxia signaling pathway, and it may regulate PCa tumorigenesis under the control of HIF-1α/SMAD3 (Figure 6E). Regulation of CDCA2 by HIF-1α was further supported by other published GEO datasets. We found CDCA2 was increased in PCaPC3 cells (GSE53012 and GSE80657) or lung cancer A549 cells (GSE48134) in the hypoxia condition (Figures 6F–H). In addition, we found that inhibition of HIF-1α could reduce *CDCA2* expression. Echinomycin, which blocks the binding of HIF-1α, repressed CDCA2 expression in glioma U251 cells (GSE7835) (Figure 6I). Knockdown of HIF-1α caused CDCA2 mRNA levels to decline in breast cancer MCF7 cells (GSE3188) (Figure 6J).

## Discussion

In our paper, we identified differential expression genes by RNA-Sequencing in PCa and found the CDCA family to be overexpressed in PCa cell lines and patients' samples. Among the CDCA family, we chose *CDCA2* for further study. A serial experiments and dataset analysis further confirmed that CDCA2 was upregulated in primary patients' samples and positively correlated with the patients' clinical stage and poor progression in prostate cancer. To our great interest, we found *CDCA2* was not only overexpressed in PCa but also upregulated in at least 18 cancer types in the TCGA dataset. What is more, higher *CDCA2* expression was correlated to patients' survival in some cancer types, such as in LAUD, KIRP, and LIHC. We further demonstrated that *CDCA2* was induced in hypoxia and directly regulated by the HIF-1α/Smad3 complex. All these suggested the potential oncogenic role of CDCA2 in PCa and other tumors.

Here, we demonstrated that inhibition of *CDCA2* induced apoptosis and repressed cellular proliferation *in vitro*. Silencing of *CDCA2* also repressed tumor growth *in vivo*. As a member of the *CDCA* family, *CDCA2* has been reported to be oncogene in several other tumors. Uchida *et al* found that CDCA2 prevented the G1 phase arrest via decreasing the expression of CDKIs and regulation of the DDR. Consistent with its function as an oncogene in OSCCs, CDCA2 is frequently upregulated in OSCCs, and overexpression of CDCA2 positively correlates with high-stage OSCCs (5). Shi et al. found that CDCA2 is widely overexpressed in lung adenocarcinoma (LAC), and a high level of CDCA2 correlates with a worse prognosis (36). Feng et al. found that CDCA2 was overexpressed in colon cancer and promotes colon cancer cell proliferation and tumorigenesis, probably through the PI3K/AKT pathway (41). Some studies have suggested that CDCA2 and PP1γ form an essential complex to recruit PP1 to chromatin, which is essential for cell growth and cell viability (19, 42). Those reports further support our finding about CDCA2 in PCa.

To elucidate CDCA2's oncogenic role in PCa, we further found that the loss of CDCA2 will increase the mRNA levels of some tumor suppressor genes, including *RASSF1, KISS1, PML*, and *GADD45A*. These tumor suppressor genes are involved in cell proliferation regulation and apoptosis (43–46); our findings will provide new insight for the mechanism of CDCA2 in tumorigenesis in addition to its regulation of CDKIs.

Hypoxia, which will cause stress-induced oxidative DNA damage, DNA strand breaks, and genetic aberrations, is a common characteristic of all solid tumors. Cancer cells, with serial genetic or epigenetic changes, can adapt to the hypoxia condition by interacting some signaling pathway (11, 47). Consequently, hypoxic tumor cells continue to proliferate, become more invasive and are usually resistant to radiotherapy and chemotherapy. The central component for hypoxia stimulation is the HIF complex (11, 47). Through exploring some public ChIP-Sequencing and RNA-Sequencing datasets, we proved that CDCA2 was induced by hypoxia condition and is involved in HIF1 signaling pathway and directly controlled by HIF-1α and SMAD3 in PCa. It was reported that HIF-1α and Smad3 interact directly and synergistically regulate VEGF and endoglin expression (17, 18). This finding indicates the potential important clinical significance of CDCA2 in PCa. The regulation of CDCA2 under the hypoxia pathway was further supported by other microarray GEO datasets, which were tested in PCa, lung cancer, glioma, and breast cancer (26–30).

Besides the direct regulation by HIF-1α and SMAD3, through some other public ChIP-Sequencing dataset, we also found that, in PCa, CDCA2 was a directly regulated by MEN1, which function as an oncogene in prostate cancer (GEO: GSE132827, Supplemental Figure 2). This is another mechanism for CDCA2 expression and further validation for the fact that binding and a new mechanism is needed in future.

## Conclusions

In conclusion, our paper found that CDCA2 is overexpressed in prostate cancer patients. It regulates cellular proliferation both *in vitro* and *in vivo* and is a direct target of the hypoxia signaling pathway in prostate cancer. Our data suggest that CDCA2 might be a potential therapeutic biomarker for prostate cancer.

## Data Availability Statement

The datasets analyzed in this study can be found in The Cancer Genome Atlas (https://portal.gdc.cancer.gov/); the NCBI Gene Expression Omnibus (GSE106305, GSE53012, GSE132827, GSE48134, GSE7835, GSE3188, GSE132827). The original contributions presented in the study can be found here: GSE148544.

## Ethics Statement

The studies involving human participants were reviewed and approved by Ethics Committee of Shenzhen People's Hospital. The patients/participants provided their written informed consent to participate in this study. The animal study was reviewed and approved by Laboratory Animal Ethics Committee of Shenzhen University.

## Author Contributions

YZ, ZZ, ZB, HJ, XG, XH, ML, and MW performed the experiments. YC, XS, YYu, and YYi performed the bioinformatics analysis of high-throughput data and the mining of public datasets. YZ, YC, XS, and YYi conceived and supervised this study and drafted the manuscript.

## Conflict of Interest

The authors declare that the research was conducted in the absence of any commercial or financial relationships that could be construed as a potential conflict of interest.
