# Supplementary material for: CDCA2 Inhibits Apoptosis and Promotes Cell Proliferation in Prostate Cancer and Is Directly Regulated by HIF-1α Pathway
Source: Front Oncol. 2020 May 19;10:725. doi: 10.3389/fonc.2020.00725 (PMC7248370; doi:10.3389/fonc.2020.00725)
Supplement: Supplementary file 1 [file Data_Sheet_1.doc]

**Supplemental table 1: list of primers** used in this study

| **Application** | **Primer name** | **Sequence (5’→3’)** | **Size (bp)** |
| --- | --- | --- | --- |
| Realtime PCR | CDCA1F | ATATTTCAGAGAAAACCAAGCGTT | 149bp |
|  | CDCA1R | CTTCTGGACCGTATCTTTCATTTT |  |
|  | CDCA2F | GCATCCGAAGACTGGGTTCA | 57bp |
|  | CDCA2R | TCACTTCTTCCAGTTTGCCA |  |
|  | CDCA3F | TTGGTATTGCACGGACACCT | 145bp |
|  | CDCA3R | GATAAAGGTGCCTCTGGGGG |  |
|  | CDCA4F | CTCCTTCCTCAGCGGCG | 116bp |
|  | CDCA4R | TTCCTCTTCAGTCCTCGTGC |  |
|  | CDCA5F | CCATGCTGTAGAGGTCCCAG | 86bp |
|  | CDCA5R | CAGGGGGCTCGTTTTCTTTC |  |
|  | GAPDHF | TCATTGACCTCAACTACATG | 131bp |
|  | GAPDHR | TCGCTCCTGGAAGATGGTGAT |  |
|  | GADD45AF | CTTGGAGACCGACGCTGG | 149bp |
|  | GADD45AR | TGTAGCGACTTTCCCGGC |  |
|  | RASSF1F | GTTCACCTGCCACTACCGC | 133bp |
|  | RASSF1R | AAGGTCAGGTGTCTCCCACT |  |
|  | KISS1F | TGGCCTCTGTGGGGAATTCTA | 104bp |
|  | KISS1R | AGCTGGCTTCCTCTCGGTG |  |
|  | PMLF | CAACATCTTCTGCTCCAACC | 73bp |
|  | PMLR | CTTGGAACATCCTCGGCAG |  |

**Supplementary table 2: Increased expression of CDCA2 in some cancer types of TCGA dataset**

| **Cancer type** | **upregulation** | **p value** |
| --- | --- | --- |
| bladder urothelial carcinoma | up | 1.58E-14 |
| Breast adenocarcinoma | up | <1E-12 |
| colon adenocarcinoma | up | <1E-12 |
| lung adenocarcinoma | up | 1.62E-12 |
| kidney renal clear cell carcinoma | up | <1E-12 |
| prostate adenocarcinoma | up | 1.03E-04 |
| Lung squamous cell carcinoma | up | 1.62E-12 |
| HNSCC | up | 1.62E-12 |
| Kidney renal papillary carcinoma | up | 2.62E-07 |
| esophageal carcinoma | up | <1E-12 |
| liver hepatocellular carcinoma | up | 1.62E-12 |
| cervical squamous cell carcinoma | up | 1.62E-12 |
| rectum adenocarcinoma | up | 1.50E-03 |
| uterine corpus endometrial carcinoma | up | <1E-12 |
| glioblastoma multiforme | up | 1.62E-12 |
| cholangiocarcinoma | up | 5.64E-08 |
| Kidney chromophobe | up | 6.40E-03 |
| stomach adenocarcinoma | up | 1.62E-12 |


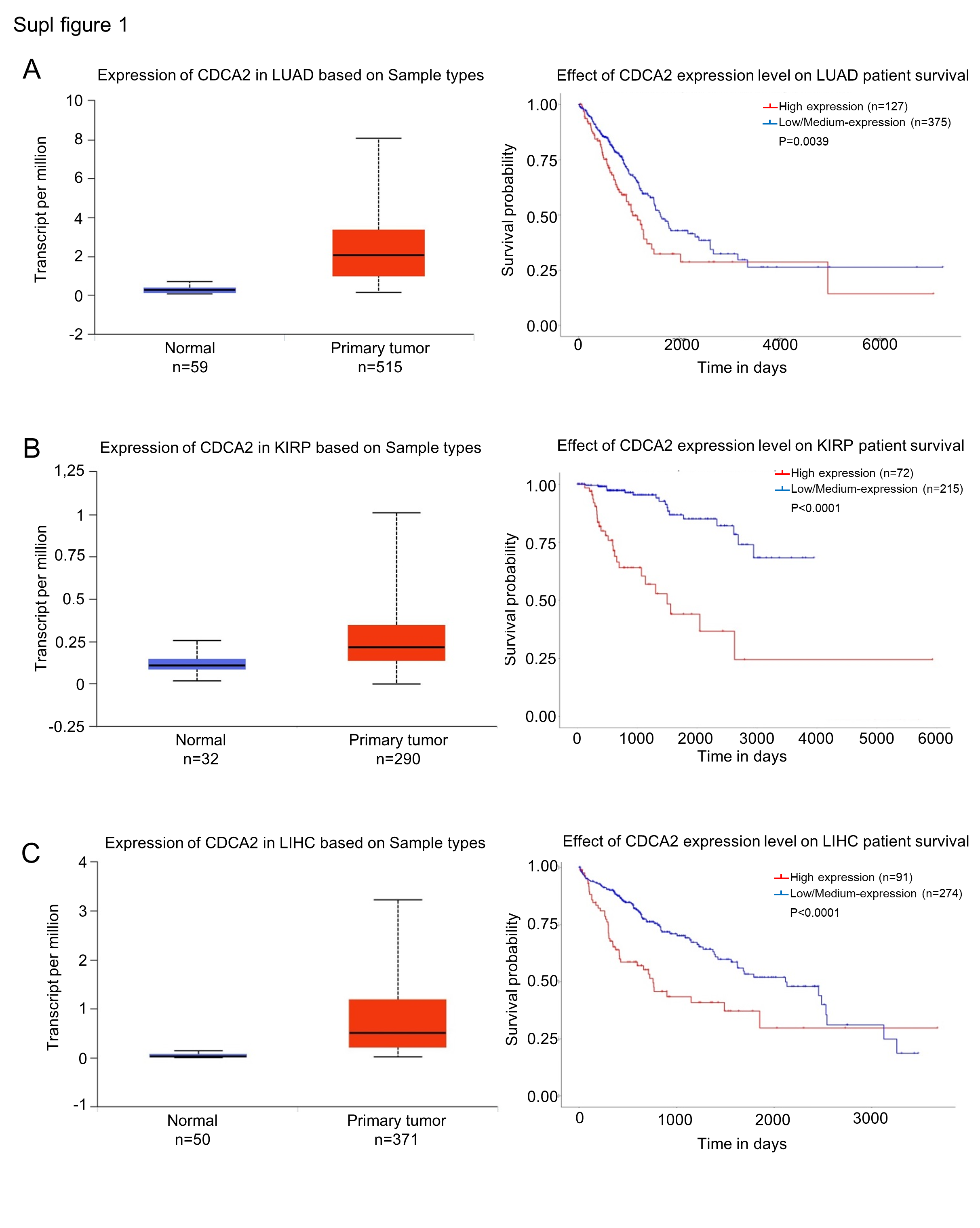


**Supplementary figure 1**: Expression level and survival data CDCA2 in TCGA datasets including LUAD (A), KIRP (B) and LIHC (C).


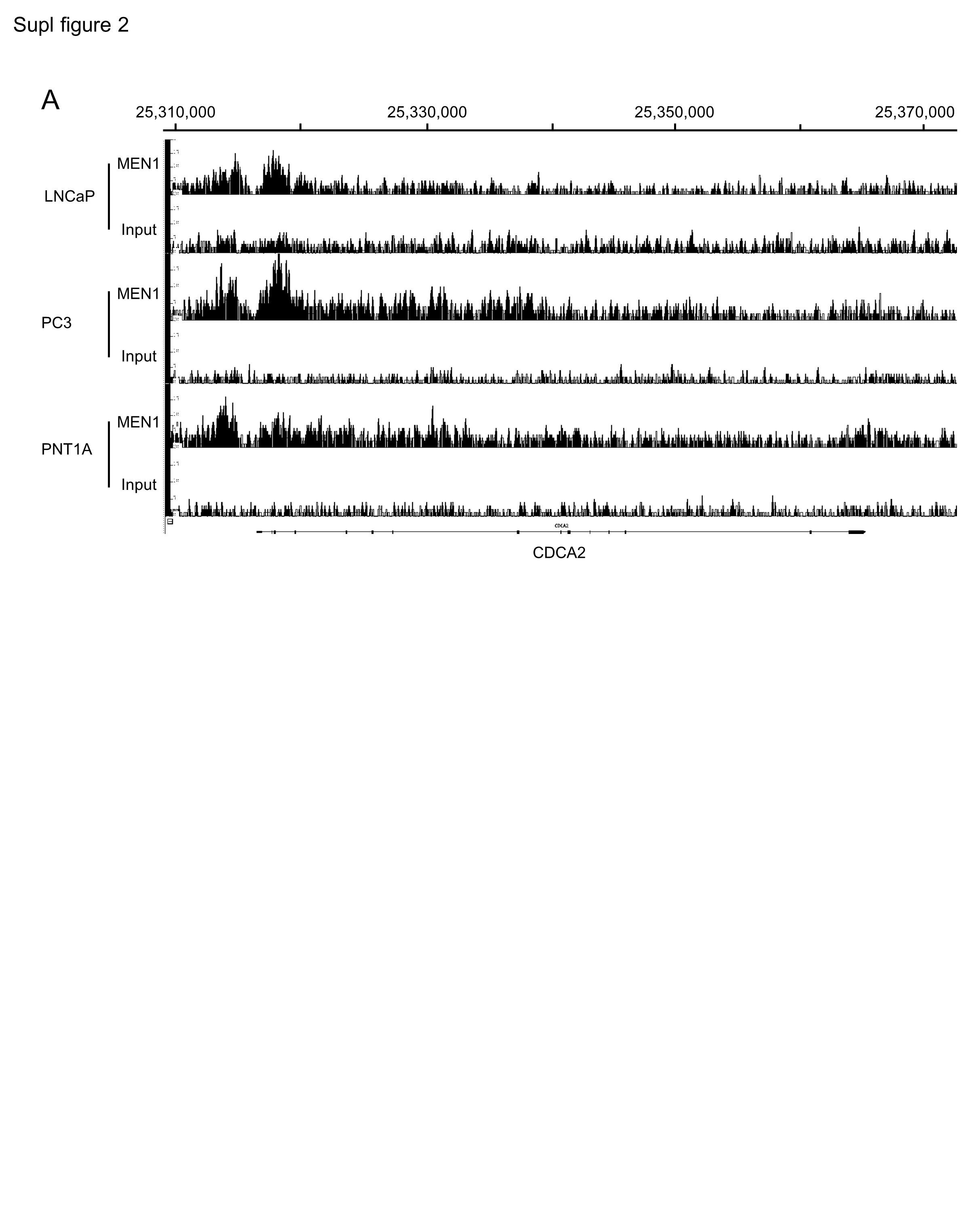


**Supplementary figure 2**: ChIP-Seq of MEN1 on CDCA2 promoter in prostate samples.
